# MPL receptor dimerization and the thrombopoietin pathway in primary immune thrombocytopenia: from molecular mechanisms to targeted therapies

**DOI:** 10.3389/fmed.2026.1798504

**Published:** 2026-06-08

**Authors:** Yujue Wang, Chengyan Liu, Yue Hu, Xiaoqi Sun, Weijie Zhang, Wenwei Zhu

**Affiliations:** 1 Department of Hematology, Yueyang Hospital of Integrated Traditional Chinese and Western Medicine, Shanghai University of Traditional Chinese Medicine, Shanghai, China; 2 First Department of Oncology, Yueyang Hospital of Integrated Traditional Chinese and Western Medicine, Shanghai University of Traditional Chinese Medicine, Shanghai, China; 3 Pharmacy Department, Yueyang Hospital of Integrated Traditional Chinese and Western Medicine, Shanghai University of Traditional Chinese Medicine, Shanghai, China

**Keywords:** primary immune thrombocytopenia, TPO receptor agonists, MPL, treatment response heterogeneity, TPO-MPL axis

## Abstract

Primary immune thrombocytopenia (ITP) is characterized by the dual pathology of peripheral immune-mediated platelet clearance and restricted platelet production by megakaryocytes. The TPO-MPL axis is a central regulator of platelet homeostasis, and ligand-induced receptor activation is associated with downstream JAK2-STAT, MAPK, and PI3K-AKT signaling. This review summarizes the structural and functional features of TPO and MPL, the major immunopathogenic mechanisms of ITP, and the current clinical use of thrombopoietin receptor agonists (TPO-RAs) in ITP. Recent structural and functional studies suggest that TPO-RAs with distinct binding sites may not engage MPL in fully identical ways and may therefore be associated with differences in receptor conformation, dimer geometry, and downstream signaling output. Within this emerging structural and functional framework, these structural and signaling differences may help explain response heterogeneity and the clinical observation that some patients may still benefit after switching agents. However, direct experimental evidence linking switching outcomes to specific alterations in MPL dimer geometry remains lacking, and the available clinical evidence regarding switching comes mainly from retrospective and observational studies, with additional support from *post hoc* analyses. Overall, continued investigation of the TPO-MPL pathway and its structural basis may help refine our understanding of ITP pathogenesis, treatment-response heterogeneity, and the clinical observations associated with switching, although the proposed mechanistic links still require direct experimental validation.

## 1 Introduction

Primary immune thrombocytopenia (ITP) is an acquired autoimmune condition of unknown etiology and is predominantly characterized by isolated peripheral blood thrombocytopenia. The predominant pathogenic factors are the immune-mediated death of peripheral blood platelets and inadequate platelet synthesis by megakaryocytes, resulting in diminished platelet numbers (1). In individuals with ITP, despite drastically decreased platelet counts, circulating thrombopoietin (TPO) levels are often normal to mildly increased and considerably lower than those observed in conditions like aplastic anemia, which is predominantly defined by megakaryocytopenia. This indicates that ITP demonstrates a biological characteristic of inadequate platelet synthesis along with diminished TPO reactivity (2). The management of ITP continues to evolve. Corticosteroids are the primary treatment; however, recent guidelines advocate for thrombopoietin receptor agonists (TPO-RAs) and rituximab for patients who do not attain remission with first-line pharmacotherapy. Numerous randomized clinical trials have confirmed the efficacy and safety of these treatments (3–6).

The varied treatment responses of TPO-RA in clinical practice necessitate further investigation. Although the immunopathogenic mechanisms of ITP and the clinical value of TPO-RAs have been widely recognized, understanding of the structural basis of the TPO-MPL axis in different treatment responses remains limited. In particular, variability in responses to TPO-RAs, together with the observation that some patients may still benefit after switching agents, suggests that MPL signaling may not be fully explained by receptor activation alone. Recent structural studies of the TPO-MPL complex provide a useful framework for reconsidering this issue in terms of receptor conformation, dimer geometry, and downstream signaling output. This review focuses on the immunopathogenic mechanisms of ITP, the structural and signaling basis of the TPO-MPL axis, and the structural characteristics and clinical applications of TPO-RAs, and further examines how these aspects may relate to treatment-response heterogeneity and the clinical observation that some patients may still benefit after switching agents. Nonetheless, these proposed connections remain largely inferential, being derived mainly from structural and functional observations, and still require validation by more direct mechanistic investigations.

## 2 Pathogenesis of ITP

ITP is a diverse immune-mediated condition characterized by thrombocytopenia resulting from two interrelated main mechanisms: markedly enhanced peripheral platelet clearance/destruction and compromised platelet synthesis (megakaryocyte maturation and release) in the bone marrow. Recent studies have enhanced the classical model from antiplatelet antibodies to FcγR-mediated phagocytosis and uncovered various simultaneous pathogenic pathways, encompassing Fc-dependent, and Fc-independent mechanisms (desialylation, apoptosis, and complement-mediated), cellular immunity, and modifications in the bone marrow immune microenvironment (7).

### 2.1 Augmented platelet degradation

In 1951, Harrington and associates hypothesized that a blood factor seen in ITP patients induced platelet loss. Upon reinjection of plasma from ITP patients, platelet counts swiftly diminished within hours, accompanied by pronounced symptoms. Following the transfusion of this plasma to several healthy hospital personnel, a temporary and notable reduction in platelet counts was noted in all instances, with levels normalizing within a few days. Bone marrow tests indicated normal megakaryocytes, demonstrating that a circulating factor in ITP patients induces platelet destruction rather than a deficiency in bone marrow hematopoietic function (8, 9). Subsequent study revealed that this circulating factor comprises a collection of immunoglobulins (10). Investigations into the pathophysiology of ITP have demonstrated that IgG autoantibodies are identified in the majority of ITP patients, specifically targeting glycoproteins GPIIb/IIIa, GPIb/IX, and GPV associated with platelets (1, 11, 12). In patients with ITP, autoantibodies targeting GPIIb/IIIa attach to the matching antigens on platelet membranes. As platelets traverse the spleen, the antibody-labeled platelets become prone to interacting with Fcγ receptors (namely FcγRI and FcγRIII) on the surface of splenic macrophages through the Fc component of the antibody. This interaction activates spleen tyrosine kinase and downstream signaling cascades, thereby promoting macrophage phagocytosis and accelerating the clearance of circulating platelets (13–15).

Research has revealed a hepatocyte-mediated, Fc-independent mechanism for platelet clearance in the liver, indicating that, in ITP, anti-GPIbα and anti-GPIb/IX antibodies can induce platelet activation, translocation of surface neuraminidase-1 (NEU1), and platelet desialylation. De-sialylated platelets are eliminated through the Ashwell-Morell receptor (AMR) located on hepatocyte membranes. This system impacts platelet clearance and regulates JAK2/STAT3, affecting hepatic TPO transcription; hence, it directly connects peripheral destruction to TPO production through feedback (16–20). Anti-GPIIb/IIIa antibodies seem to more effectively promote NEU1 surface translocation, whereas anti-GPIIb/IX complex antibodies lead to an increased incidence of platelet death (17).

The activation mechanism of the complement system in ITP patients predominantly depends on autoantibodies for complement fixation. GPIIb/IIIa and GPIb/IX are the primary autoantibody targets in ITP, and their capacity to activate complement is tightly associated with the specific antigen type targeted by the antibodies (21–23). Research suggests that antibodies directed against GPIIb/IIIa activate the complement system more effectively than those directed against GPIb/IX, which may intensify platelet clearance (23). C3b, formed on platelet surfaces after complement activation, can be identified by macrophage complement receptors (e.g., CR1). This recognition complements traditional FcγR signaling, markedly improving the phagocytic clearance efficiency of splenic macrophages and further intensifying thrombocytopenia (24).

Multidimensional analysis indicates an increase in terminal effector memory CD8^+^T cell (TEMRA) clones in patients with chronic ITP. These cells exhibit elevated expression of IFN-γ, TNF-α, and granzyme B, demonstrating persistent cytotoxic activity devoid of exhaustion signals. TEMRA cells consistently proliferate when platelet levels diminish (25). Additionally, CD8^+^T lymphocytes from patients with ITP establish persistent aggregates with autologous platelets. These aggregates cause platelet activation and death by TCR-mediated release of cytotoxic granules containing interferon-γ (25).

### 2.2 Impaired platelet production

In addition to heightened peripheral platelet breakdown, compromised megakaryocyte activity and inadequate thrombopoiesis are fundamental pathogenic elements of ITP. Platelets are generated from mature megakaryocytes through the extension and remodeling of proplatelet processes (26–28). Studies have shown that antiplatelet autoantibodies in ITP inhibit proplatelet formation by megakaryocytes and thereby impair platelet production *in vitro* (29). In pertinent *in vitro* research, CD34^+^ cells from healthy donors were grown in a solution comprising pegylated recombinant human megakaryocyte growth and development factor (PEG-rHuMGDF) and 10% plasma from either ITP patients or healthy individuals. In comparison to healthy control plasma, plasma from 12 of 18 ITP patients significantly inhibited *in vitro* megakaryocyte generation; the positive ITP plasma not only diminished the total number of megakaryocytes produced during culture but also hindered megakaryocyte maturation, evidenced by a reduction in 4N, 8N, and 16N megakaryocytes (30). Two human monoclonal autoantibodies derived from ITP patients−2E7, targeting the heavy chain of human platelet glycoprotein IIb, and 5E5, directed against a novel antigen on glycoprotein IIIa present on activated platelets—demonstrated a substantial inhibitory effect on *in vitro* megakaryopoiesis (*P* < 0.001) (31). Bone marrow examination in individuals with ITP generally shows normal or elevated megakaryocyte numbers (32).

*In vitro* studies investigating the effects of plasma from patients with ITP on megakaryocyte apoptosis showed that most ITP plasma samples inhibit megakaryocyte apoptosis, resulting in an increased number of megakaryocytes but reduced megakaryocyte quality and impaired thrombopoiesis. Simultaneously, the expression of tumor necrosis factor-related apoptosis-inducing ligand (TRAIL) was diminished, whilst the expression of the anti-apoptotic factor Bcl-xL was heightened. Abnormal megakaryocyte apoptosis may lead to diminished peripheral blood platelet counts in patients with ITP (33). Subsequent research suggests that this plasma-mediated inhibition of apoptosis is likely strongly associated with components derived from plasma exosomes. ITP plasma-derived exosomes also disrupt megakaryocyte apoptosis via the Bcl-xL/caspase signaling pathway, uncovering a unique non-cell-autonomous regulation mechanism that results in diminished thrombopoiesis (34). Moreover, single-cell research focusing on hematopoietic origin demonstrated that CD9^+^ hematopoietic stem/progenitor cells (HSPCs) in ITP patients not only diminished in quantity but also displayed an altered differentiation propensity from the megakaryocytic to the erythroid lineage. This led to markedly diminished differentiation potential toward megakaryocytes, intensifying the crisis in platelet production upstream (35).

Besides heightened platelet breakdown in the peripheral circulation, ITP may also correlate with compromised megakaryocyte maturation and diminished preplatelet production. Antiplatelet autoantibodies in ITP patients can directly obstruct preplatelet production in megakaryocytes and hinder platelet release (29, 36). In ITP, circulating TPO levels do not usually increase commensurately with the reduction in platelet count and are generally lower than in thrombocytopenic conditions largely resulting from megakaryocyte shortage, implying that TPO-mediated compensation is not entirely efficient in ITP (37, 38). Moreover, anti-c-Mpl autoantibodies have been detected in a subset of patients with ITP; this subgroup is characterized by higher serum TPO levels, lower megakaryocyte and platelet counts, and reduced responsiveness to rhTPO treatment (39). Consequently, regarding platelet production in the bone marrow, ITP is characterized not only by the heightened destruction of peripheral blood platelets but also by compromised megakaryocyte maturation and platelet production, inadequate TPO compensation, and possible receptor-level anomalies in certain patients (36, 37, 39).

## 3 Molecular basis of the TPO/MPL signaling axis

### 3.1 TPO architecture and physiological role

TPO is predominantly expressed in the liver as a precursor protein with a molecular weight of approximately 36 kDa and consisting of 353 amino acids (40–42). The resultant 332-amino acid secretory protein, following the excision of a 21-amino acid signal peptide, undergoes glycosylation changes. This produces a glycoprotein that manifests at around 95 kDa on SDS-PAGE and is quantified at approximately 57.5 kDa by mass spectrometry (43, 44). TPO consists of two domains: the N-terminus features two conserved disulfide bridges, whilst the C-terminus regulates TPO half-life and contains glycosylation regions linked to secretion (45). The N-terminal region exhibits similarity with 153 residues of human erythropoietin (EPO); nevertheless, EPO and MPL do not compete for binding to their respective receptors (46). The C-terminal region is extensively glycosylated, having a negligible effect on TPO stability and signal transmission (40, 41, 47–49).

**Figure 1:**
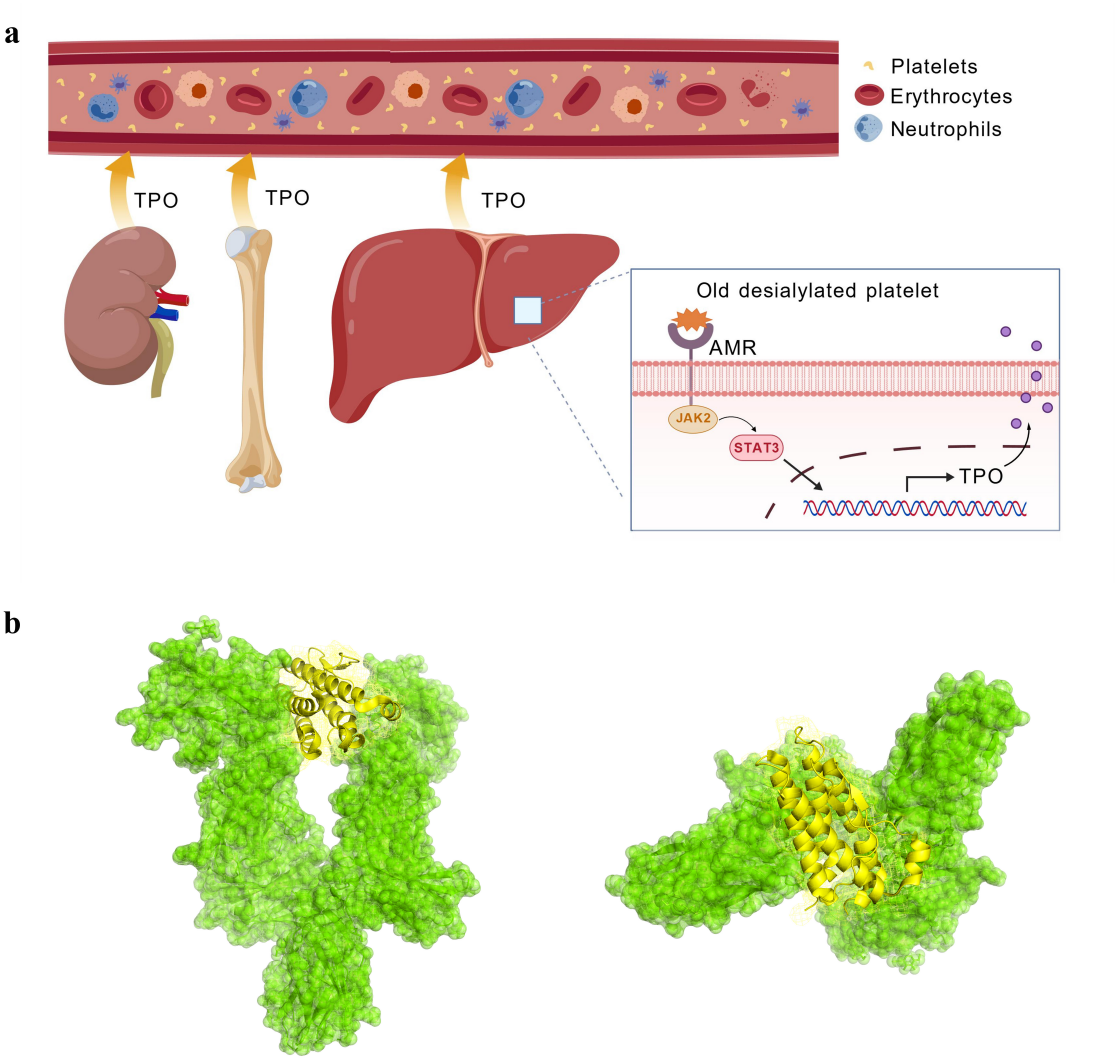
**(a)** The liver is the principal site of TPO production, while the kidneys and bone marrow also contribute minor quantities of TPO. Upon entering circulation, TPO exerts its effects on hematopoietic stem/progenitor cells and the megakaryocyte-platelet lineage. Senescent, de-sialylated platelets are identified and eliminated by AMR on hepatocyte membranes, while triggering the JAK2-STAT3 signaling cascade. This enhances TPO gene transcription in hepatocytes, establishing a feedback regulatory loop. **(b)** TPO functions in conjunction with MPL.

TPO is a crucial regulator in thrombopoiesis, significantly impacting the entire process from the proliferation and differentiation of hematopoietic stem cells into megakaryocytes to the maturation and ultimate release of functional platelets, affecting practically all facets of platelet formation (50). Endogenous TPO is predominantly produced in the liver, with modest contributions from the bone marrow and kidneys, and is immediately released into the peripheral bloodstream (51, 52). Researchers have found that, under standard physiological settings, TPO levels in blood and bone marrow exhibit an inverse correlation with platelet counts (51). The regulation of circulating TPO involves various mechanisms, with the identification and removal of desialylated platelets by the hepatic AMR being a crucial feedback loop that influences hepatic TPO production; this process activates the JAK/STAT pathway, facilitating hepatic TPO transcription and synthesis (50, 53). Moreover, research demonstrates that TPO has anti-apoptotic effects in both the early and late phases of megakaryocyte formation (54, 55) (Figure 1a).

### 3.2 MPL architecture and physiological role

The myeloproliferative leukemia protein (MPL), commonly referred to as the TPO receptor, is a type I cytokine receptor found in hematopoietic stem cells and the megakaryocyte lineage. It facilitates development and differentiation into the megakaryocyte lineage and is crucial for optimal platelet production (56–59). MPL is a protein consisting of 635 amino acids, encoded by the c-Mpl gene, and includes an extracellular domain (ECD), transmembrane helices (TM), and an intracellular domain (ICD). The ECD comprises two cytokine receptor-like modules (CRMs), identified as CRM1 and CRM2 (60). CRM1 mediates TPO recognition, whereas CRM2 is essential for downstream signaling (61). CRM1 is partitioned into D1 and D2 domains, while CRM2 is separated into D3 and D4 domains (62). Moreover, a disulfide bond links the D2 and D3 domains of MPL, reinforcing the structural alignment between CRM1 and CRM2. This covalent interaction likely stabilizes their spatial configuration, which may be essential for transmitting the ligand-induced conformational changes toward the membrane-proximal regions. Such an arrangement could facilitate the close positioning of the D4 domains, a prerequisite for effective homodimerization and activation of MPL (61, 63–66).

### 3.3 Structural foundation of the TPO-MPL signaling complex

The TPO-MPL signaling pathway is crucial for sustaining platelet homeostasis, and its dysregulation results in several hematological diseases, such as ITP and aplastic anemia. Recent cryo-electron microscopy studies have elucidated the molecular structure of the TPO-MPL complex (Figure 1b). TPO links two MPL chains through the high-affinity site 1 and low-affinity site 2 interface, establishing a TPO-specific binding configuration (61).

When ligand occupancy is limited, surface MPL is thought to exist predominantly as monomers with low basal signaling, while a smaller fraction may associate with TPO through the high-affinity interface to form a dimeric non-signaling complex (MPL:TPO 1:1), which may represent a pre-signaling assembly state (48, 49, 56, 60, 61). With an increase in TPO concentration, the proportion of dimeric complexes on the cell surface escalates (60). A signaling-competent ternary complex (MPL:TPO 2:1) is established through a low-affinity interface with an additional MPL molecule, thereby establishing a distinct dimeric configuration that is associated with JAK2 cross-activation and subsequent signaling output (48, 49, 56, 61). The high-affinity binding site 1 is established through interactions between residues on D1 and D2 within the CRM1 domain and the α-helix B of TPO (61). This ternary complex is completed through site 2, which is established by interactions between MPL D1 and TPO helix C, as well as between MPL D2 and TPO helix A (61). The high-affinity site is thought to facilitate initial receptor engagement, whereas the low-affinity site may help stabilize a signaling-competent receptor arrangement across the membrane.

### 3.4 Downstream signaling cascades

**Figure 2:**
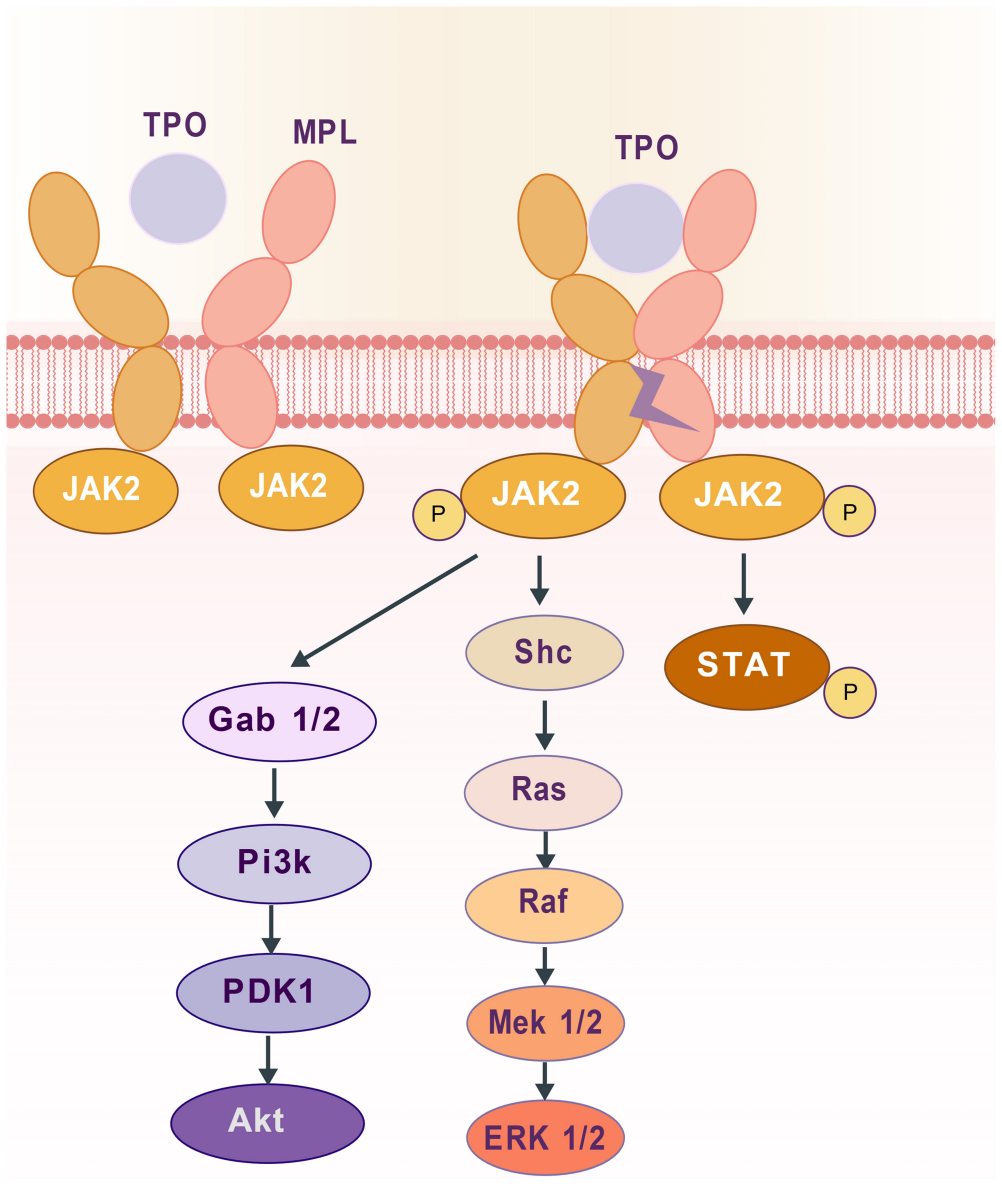
Schematic representation of the JAK2 downstream signaling pathway mediated by the TPO/MPL complex. TPO interacts with its receptor MPL, prompting receptor dimerization and activating transmembrane JAK2. The autophosphorylated JAK2 subsequently phosphorylates and recruits adaptor proteins, including Shc and Gab1/2. It triggers the ERK1/2 cascade through the Shc-Ras-Raf-Mek1/2 pathway, while simultaneously activating the Akt pathway via the Gab1/2-PI3K-PDK1 pathway. Concurrently, JAK2 directly phosphorylates STAT proteins, facilitating their dimerization and nuclear translocation to modulate target gene transcription. These systems collectively regulate megakaryocyte proliferation, differentiation, platelet formation, and survival.

The interaction of TPO with MPL triggers conformational alterations and dimerization of the receptor, thereby activating JAK2 linked to its intracellular domain. JAK2 phosphorylation is a central event in signal initiation. Activated JAK2 subsequently phosphorylates tyrosine residues within the MPL intracellular domain and recruits downstream signaling molecules, thereby initiating a signaling network centered on three major pathways: JAK-STAT, RAS-MAPK, and PI3K-AKT. This network contributes to MPL stability, cell-surface expression, and TPO-induced signal transduction (53, 67–72) (Figure 2). The JAK-STAT pathways primarily facilitate proliferation, but MAPK and PI3K-AKT demonstrate stage-dependent variations in differentiation, survival, and platelet formation. Consequently, rather than viewing JAK-STAT, MAPK, and PI3K-AKT as fully independent pathways, it may be more appropriate to consider them as interconnected signaling networks responsive to receptor geometry and signaling context, which may help explain variability in cellular responses to MPL activation (68).

### 3.5 MPL dimer geometry and signal bias: from structure to testable hypotheses

Signal bias refers to differential activation of STAT, ERK, and AKT signaling and the resulting differences in cellular outcomes elicited by different agonists during receptor activation (59, 73, 74). This concept may help interpret response heterogeneity at the cellular and pharmacologic levels by relating it to receptor dimer geometry, receptor-kinase abundance, and stage-specific sensitivity of downstream networks (62, 68, 72). In the Class I receptor family, the relative rotation and spacing of transmembrane helices modify intracellular JAK location and the probability of cross-activation. Consequently, dimer geometry may function as an intermediary variable converting ligand binding into enzymatic activation probability (61–64).

Potential differences in receptor geometry among TPO-RAs are plausibly related to their distinct binding sites (59, 75). TPO binds the extracellular domain of MPL to assemble the signaling complex, and peptide agonists such as romiplostim likewise act through the extracellular domain of the receptor, in contrast to small-molecule agonists that engage the juxtamembrane and transmembrane regions (59, 75). By contrast, small-molecule agonists such as eltrombopag engage the juxtamembrane and transmembrane regions in a noncompetitive manner with TPO and may therefore influence receptor activation through a mode of receptor engagement distinct from that of extracellular-domain agonists. This distinct mode of engagement may contribute to differences in downstream signaling output (75–77). However, whether such differences directly account for the continued clinical benefit observed after switching agents remains unproven (59, 74, 75).

## 4 Thrombopoietin receptor agonists: structural characteristics and clinical applications in ITP

### 4.1 Current TPO-RAs: distinct modes of MPL engagement

TPO-RAs have been included in essential second-line regimens for adult ITP in consensus statements and guidelines and are extensively utilized for long-term maintenance and therapy (3–6). TPO-RAs can be broadly classified as ECD agonists, including rhTPO and romiplostim, and TM agonists, including eltrombopag, avatrombopag, and hetrombopag (55, 76). Clinical observations suggest that these agents are not always interchangeable, and some patients may still benefit after switching between different TPO-RAs (78).

**Figure 3:**
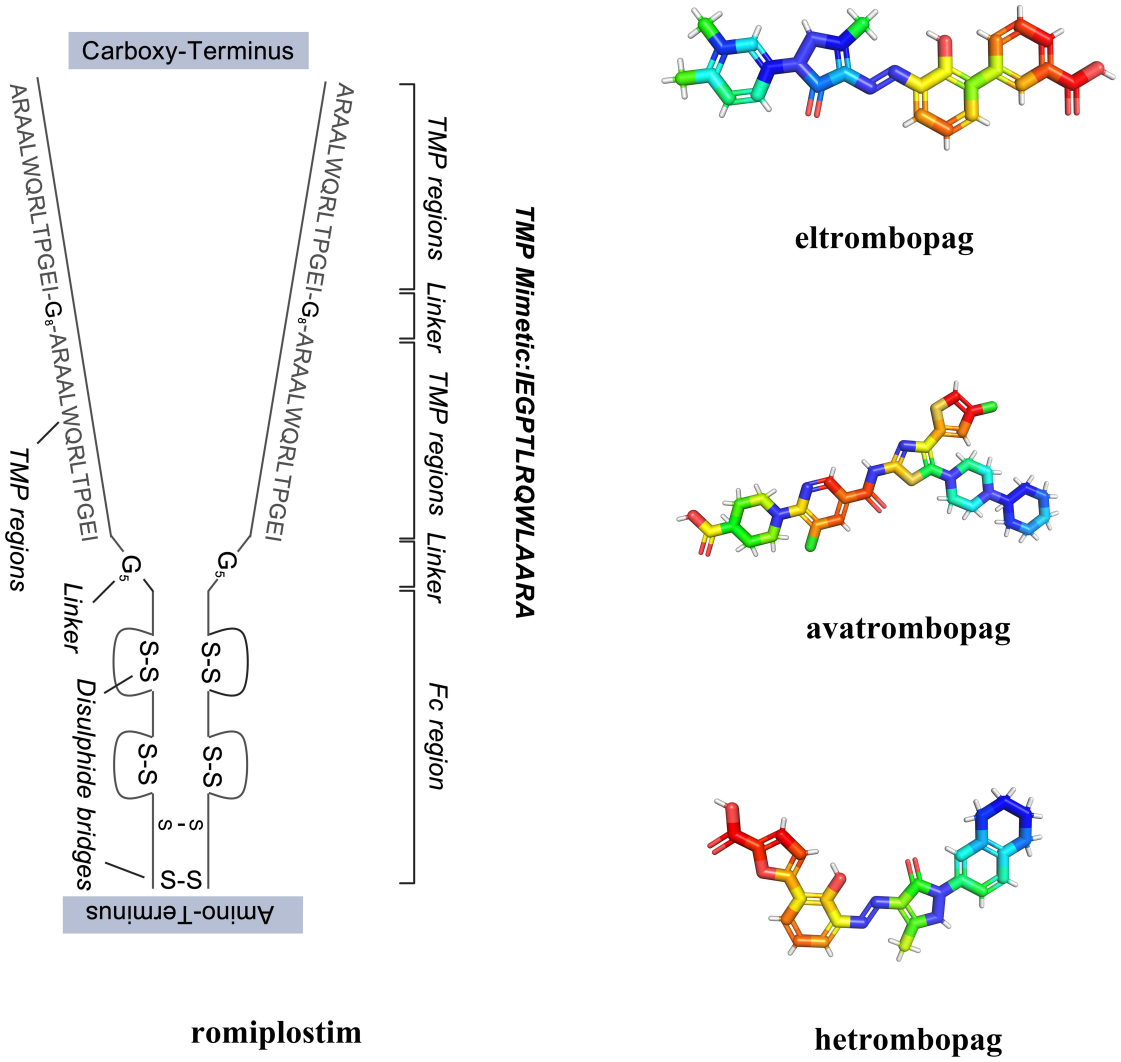
Structures of romiplostim (103), eltrombopag, avatrombopag, and hetrombopag. The molecular depictions of eltrombopag, avatrombopag, and hetrombopag were generated from PubChem.

The initially developed exogenous TPO-like agents include rhTPO and PEG-rHuMGDF (76, 79). rhTPO has the same amino acid sequence as endogenous TPO and is currently recommended as a second-line therapy for ITP in the Chinese treatment guidelines (79, 80). Subsequent studies demonstrated that PEG-rHuMGDF stimulates the generation of autoantibodies against PEG-rHuMGDF in both patients and healthy individuals. These antibodies exhibit cross-reactivity with endogenous thrombopoietin, resulting in thrombocytopenia (43, 81–84). Among second-generation TPO-RAs, romiplostim acts as an extracellular-domain agonist, whereas eltrombopag, avatrombopag, and hetrombopag are small-molecule agonists that target the transmembrane domain (Figure 3). These agents all activate MPL, but they bind to different regions of the receptor (52, 75).

Extracellular-domain agonists and juxtamembrane/transmembrane-region agonists activate MPL through different receptor-contact sites. This distinction is mechanistically relevant because receptor orientation and transmembrane helix interactions influence MPL signaling, although direct comparative structural data for currently used TPO-RAs remain limited (59, 61, 62, 85). Romiplostim displays TPO-mimetic peptides through its Fc framework to stimulate the extracellular domain of MPL and activates the receptor through extracellular-domain engagement, despite the absence of sequence homology with native TPO (76, 81, 86–91). Eltrombopag (SB497115, Promacta) activates human MPL through a mechanism that depends on the juxtamembrane and transmembrane regions, with His499 identified as a key residue required for receptor activation (77, 92). In addition, His499 has been implicated in the regulation of human TPO receptor activation and in protection against activating transmembrane mutations (77, 85). Clinical observations further suggest that TPO-RAs are not always interchangeable; in adults with ITP, platelet responses have been reported after switching from eltrombopag or romiplostim to avatrombopag (78, 93, 94).

Separately, functional studies have revealed unique signaling characteristics among several TPO-RAs. Eltrombopag has been reported to activate STAT/AKT/ERK signaling in association with megakaryocyte differentiation and platelet production, and its signaling profile may differ from that described for romiplostim (73, 95). Related results indicate that various TPO-RAs can elicit distinct phosphorylation responses within the same biological system. In a human megakaryocyte model, eltrombopag stimulated AKT and ERK1/2 activation linked to platelet production, exhibiting a mechanism of action that was not entirely congruent with that of rHuTPO (standard control) in the same system (73). In a comparative study, rhTPO preferentially induced pSTAT3, pAKT, and higher levels of pSTAT5, whereas eltrombopag had no apparent effect on pSTAT3, supporting the view that eltrombopag does not fully recapitulate endogenous TPO signaling and may exhibit a signaling profile distinct from rhTPO (96). Variations in residues within the juxtamembrane and transmembrane regions can alter the activity of small-molecule agonists, supporting the importance of these regions in receptor engagement (77, 92). Recent findings in structural biology, transmembrane binding sites, and functional studies indicate that various TPO-RA variants may affect MPL receptor conformation and downstream phosphorylation patterns via distinct binding sites. This variation may correlate with megakaryocyte maturation, preplatelet formation, and peripheral platelet production; however, a causal relationship requires validation through more direct mechanistic investigations (59, 62).

### 4.2 Practical use of TPO-RAs in current treatment algorithms

In adult ITP, TPO-RAs are an important second-line option after failure of first-line corticosteroids or in corticosteroid-dependent disease, and treatment selection should be individualized according to bleeding risk, disease duration, comorbidities, and patient preference (4–6). In practice, TPO-RAs are particularly useful when a non-immunosuppressive strategy is preferred, when sustained platelet support is needed, or when splenectomy is to be delayed or avoided (4–6, 97). From a practical standpoint, romiplostim may be useful when close dose titration and regular supervision are needed, or when adherence to daily oral therapy is uncertain; however, the requirement for weekly subcutaneous administration may limit its acceptability in some patients (5, 98). Eltrombopag provides an oral alternative, but its use requires caution regarding dietary restrictions, interactions with polyvalent cations, and regular liver-function monitoring, which may be particularly relevant in patients with hepatic comorbidity or complex dietary or medication schedules (5, 52, 98). Avatrombopag is also administered orally and does not require fasting or dietary modification, which may offer practical advantages for selected patients in terms of convenience and adherence (98).

In clinical practice, safety considerations influence both agent selection and monitoring strategies (97, 99, 100). Although TPO-RAs are generally well tolerated, important adverse effects to consider include thromboembolic events, eltrombopag-associated hepatotoxicity, rebound thrombocytopenia after discontinuation, and bone marrow reticulin deposition in a minority of patients (52, 97, 98). Because ITP itself is associated with an increased risk of both arterial and venous thrombosis, thrombotic events arising during TPO-RA therapy should be interpreted in the context of the underlying disease and individual patient characteristics rather than attributed solely to drug exposure (99). Accordingly, risk stratification should incorporate prior thrombosis, age, splenectomy status, antiphospholipid antibodies, and cardiovascular comorbidities when weighing the overall clinical benefit of TPO-RA therapy (99, 100).

The therapeutic goal of TPO-RA treatment is to maintain a platelet count sufficient to prevent clinically relevant bleeding rather than to normalize the platelet count, thereby balancing hemorrhagic and thrombotic risk (5, 6, 100). Taken together, the choice among TPO-RAs in routine practice is shaped not only by efficacy but also by route of administration, monitoring requirements, safety profile, and patient-specific circumstances; these agents therefore should not be assumed to be interchangeable in every clinical context (4–6, 98, 100). Within this long-term management framework, issues such as dose adjustment, monitoring burden, treatment tapering, and, in selected patients, switching between TPO-RAs become increasingly important (4, 99).

### 4.3 Long-term management of TPO-RAs in ITP: tapering, SROT, and switching

For selected patients with ITP who achieve a stable response, particularly sustained complete remission, gradual tapering and, in some cases, discontinuation of TPO-RA therapy have become clinically relevant components of long-term management (89, 101). This approach is not suitable for all patients and requires individualized assessment of response stability, platelet counts, and the overall clinical context (89, 101). Prospective studies indicate that a subset of patients can maintain sustained remission off therapy (SROT) after TPO-RA withdrawal, although robust clinical predictors of successful discontinuation remain lacking (89, 101). In one prospective romiplostim tapering study, the 1-year probability of SROT was 23.6%, suggesting that treatment-free remission can be achieved in a proportion of adults with chronic ITP (101). Immune and platelet kinetic changes observed during tapering studies suggest that durable remission after discontinuation may be associated with immunomodulatory and platelet-survival effects, although the underlying mechanisms remain incompletely understood (101).

Unlike SROT, which concerns maintenance of response after treatment withdrawal, switching between TPO-RAs is primarily a clinical management strategy used when prior therapy provides insufficient response, poor tolerability, or unstable platelet control (78, 91, 93). Reviews and real-world data indicate that TPO-RAs are broadly effective, but substantial inter-patient variability remains in response, durability, and tolerability (97). Importantly, the available evidence for switching comes mainly from retrospective cohort studies and observational analyses, with additional support from *post hoc* analyses (78, 91, 93, 94, 102). The main reasons for switching between TPO-RAs and the reported outcomes after switching in representative clinical studies are summarized in Table 1.

**Table 1:** Reasons for and outcomes of switching between TPO receptor agonists in patients with ITP.

References	1st TPO-RA	2nd TPO-RA	Reason for switching	*n*	Outcome after switch	Result (responders/total, %)
Khellaf et al. (91)	Romiplostim	Eltrombopag	Treatment failure	13	Response rate	6/13 (46)
			Platelet count fluctuation	11	Platelet-count stabilized	6/11 (55)
			Side effect	3	Good tolerance	3/3 (100)
			Patient's preference	8	-	-
	Eltrombopag	Romiplostim	Treatment failure	10	Response rate	8/10 (80)
			Side effect	1	Good tolerance	1/1 (100)
Cantoni et al. (78)	Romiplostim	Eltrombopag	Treatment failure	24	Response achieved	8/24 (33.3)
			Response loss	15	Response regained	13/15 (86.6)
			Platelet count fluctuation	9	Response maintained	7/9 (77.7)
			Patient's preference	8	5/8 (62.5)
			Side effects	3	3/3 (100)
	Eltrombopag	Romiplostim	Treatment failure	27	Response achieved	16/27 (59.3)
			Response loss	5	Response regained	3/5 (60)
			Platelet count fluctuation	2	Response maintained	2/2 (100)
			Patient's preference	0		n.a.
			Side effects	13		11/13 (84.6)
Al-Samkari et al. (93, 94)	Romiplostim	Avatrombopag	Effectiveness; convenience; adverse event	33	Platelet response (PLT ≥ 50 × 10^9^/L) and complete platelet response (PLT ≥ 100 × 10^9^/L), each achieved at least once on AVA without rescue therapy	Platelet response: 41/44 (93); complete response: 38/44 (86)
	Eltrombopag			10		
	Romiplostim/Eltrombopag			1		
	Romiplostim	Avatrombopag	Effectiveness	10		Platelet response: 12/14 (86); complete response: 10/14 (71)
	Eltrombopag			4		
	Romiplostim	Avatrombopag	Convenience	21		Platelet response: 23/23 (100); complete response: 22/23 (96)
	Eltrombopag			1		
	Romiplostim/Eltrombopag			1		
	Romiplostim	Avatrombopag	Adverse event	2		Platelet response (PLT ≥ 50 × 10^9^/L): 6/7 (86); complete response (PLT ≥ 100 × 10^9^/L): 6/7 (86)
	Eltrombopag			5		
Mei et al. (102)	Eltrombopag	Hetrombopag	Completed 14 weeks of eltrombopag treatment and switched to 24 weeks of hetrombopag	63	Treatment response, defined as ≥1; PLT ≥ 50 × 10^9^/L after switching	56/63(88.9)

In a retrospective study of 46 patients with ITP who sequentially received romiplostim and eltrombopag, switching between the two agents improved platelet counts in 50%−80% of patients, reduced platelet-count fluctuations in 54%, and resolved intolerance-related adverse effects, supporting switching as a clinically relevant option in selected patients (91). A retrospective analysis of a larger cohort switching between eltrombopag and romiplostim showed that 65% of patients achieved or maintained a short-term response, including 57.8% of those who switched because of insufficient efficacy of prior therapy (78). In a multicentre retrospective observational study, adults with ITP who had switched from eltrombopag or romiplostim to avatrombopag were evaluated (93, 94). Overall, 41/44 patients (93%) achieved a platelet response (≥50 × 10^9^/L), and 38/44 (86%) achieved a complete response (≥100 × 10^9^/L) after switching to avatrombopag (93). Among the 14 patients who switched because of insufficient effectiveness of prior TPO-RA therapy, 12/14 (86%) achieved a platelet response and 10/14 (71%) achieved a complete response, suggesting that avatrombopag may remain effective in some patients with inadequate response to a prior TPO-RA (93). Subsequent observations within the same cohort suggested that responses could be sustained over time, although the observational nature of the study does not exclude the influence of clinical or temporal confounders (94). In a *post-hoc* analysis of a multicenter randomized phase III trial, the response rate increased from 66.7 to 88.9% after switching from eltrombopag to hetrombopag, and treatment-related adverse events were also reduced (102). Because switching was not the prespecified comparative objective of that study, these findings are best interpreted as exploratory rather than definitive (102).

Taken together, these studies suggest that lack of efficacy or tolerability with one TPO-RA does not necessarily preclude benefit from another (78, 91, 93, 102). However, these clinical observations do not by themselves establish the mechanism underlying such differences (61, 62). Differences among TPO-RAs in binding sites, receptor activation modes, and downstream signaling profiles may be relevant to the biological interpretation of these clinical observations. However, current clinical evidence remains insufficient to directly link switching outcomes or response heterogeneity to specific alterations in MPL dimer geometry (61, 62).

## 5 Conclusion

ITP is not attributable to a singular cause; instead, it results from a multifactorial process involving increased peripheral immune-mediated platelet clearance and impaired platelet production, with evidence in some patients of limited compensatory TPO responses and possible abnormalities at the MPL level. As understanding of the TPO-MPL complex, receptor dimerization, and downstream signaling networks continues to evolve, MPL in ITP can also be viewed in terms of receptor conformation, dimer geometry, and signaling output, rather than receptor activation alone.

Although multiple TPO-RAs target MPL, their binding sites, modes of receptor engagement, and downstream signaling profiles are not identical. Existing structural and functional studies suggest that different TPO-RA compounds may not engage MPL in fully equivalent ways at the receptor-signaling level; however, direct evidence linking these differences to specific clinical outcomes after switching remains limited. Taken together, the available structural and functional evidence may help interpret response variability and the clinical observation of benefit after switching agents, although the underlying mechanistic links remain to be established.
